# Diseased Temporal Bone

**Published:** 1851-07

**Authors:** 


### ARTICLE XVI.

### *Diseased Temporal Bone.*

A WOMAN, aged twenty, was admitted into the free hospital in March. She had great fluttering and irregular vibratory action of the heart, resembling erethismus mercurialis, but which subsided in a day or two. She was deaf, and had long suffered from intense ear-ache, with occasionally fetid discharges from the meatus. She was restless, sleepless and occasionally delirious, with no appetite. Soon after admission, an abscess formed just above the left collar bone, which discharged large quantities of matter until her death. The disturbance of the heart's action returned after three doses of gray powder but again subsided in about two days. She then had severe delirium, which abated after a sudden, large and very fetid discharge from the left ear. Finally, she had erysipelas, violent delirium, and coma, and died April 5.

A large excavated abscess, with sinuses in various directions, was exposed at the root of the neck, communicating with and extending through the whole of the carotid sheath. The internal jugular vein was full of matter, which was also found burrowing down in the direction of the vena innominata. A fibrinous clot was found in that vein, extending to the cava, which under the microscope was found to contain pus-globules. The lungs were filled with a frothy and purulent infiltration without consolidation; there was a small circumscribed abscess between the pleura pulmonalis and the lung on the right side, but not extending into the substance of the latter. The heart healthy; liver pale, and contained numerous fat cells; the kidneys coarse; the cerebrum was healthy; the arachnoid membrane appeared in parts smeared over with pus, more particularly in the posterior part near the falx, joining the tentorium. The tentorium covering the left lobe of the cerebellum was much inflamed, thickened and had matter between it and the arachnoid covering the cerebellum; and immediately beneath this, on cutting into the cerebellum, a circumscribed abscess, the size of a walnut, was discovered. This was nearer the falx than the outer margin of the cerebellum; that part of the cerebellum which was in contact with the cranial bones was healthy. The petrous portion of the temporal bone was examined by Mr. Toynbee, whose notes are appended. The lateral sinus of that side was more conspicuous than the opposite one, shining through the dura mater, and darker colored, marking out the continuity of the abscess of the cerebellum with the disease in the bone, and with that in the carotid sheath. Dr. Heale considered that the prominent points were, that the palpitation was probably occasioned by the morbid stimulus of the pus in the blood; that the phlebitis was caused either directly by the disease in the lateral sulcus, or else propagated from the abscess in the cerebellum; that the abscess in the cerebellum was not occasioned by direct contiguity with the diseased bone, since healthy structure intervened, but was continuous with it in a tortuous direction by means of the lateral sinus; and that the cases of the accession of sleep in the cerebrum or cerebellum were secondary to disease in the auditory apparatus without the intervention of phlebitis either of the veins or of the sinuses.

*Mr. Toynbee's Notes.*—"The external meatus contained purulent matter; the glandular and periosteal portions of the membranous meatus were much softer than natural, and they adhered but slightly to the surface of the bone. The bone forming the upper and outer half of the tube was observed to present numerous foramina for the transmission of blood-vessels, much larger than in the normal state, and some of them surrounded by delicate layers of new bone; through the larger of these foramina small bristles could be passed, and they appeared to communicate with canals in the interior of the bone, which were continuous with orifices in the sulcus lateralis at its inner surface. The lateral sinus was of a dark brown color; the dura mater forming its posterior wall was entire. The sinus was full of coagulated blood mixed with purulent matter; the dura mater constituting its anterior wall, and which was in contact with the surface of the bone forming the sulcus lateralis, was very thick and soft ; portions of it were destroyed by ulceration, and the bone exposed. The bone forming the sulcus lateralis was of a dark color, and covered by masses of lymph and pus ; its surface was rough, presenting throughout numerous orifices and tortuous grooves, this appearance being produced by the almost entire disappearance of the internal table of the skull, which (excepting two scales, each measuring about two lines in diameter) had been destroyed by caries. The destruction of bone by caries had advanced so far at one point, that an orifice about a line in diameter had been produced, which communicated with the upper part of the mastoid cells. The caries was not confined to the interior of the skull, the surface of the jugular process of the occipital bone having been destroyed. There was an orifice in the posterior part of the membrana tympani, through which, probably, the virus had been discharged, as it was not found in the tympanic cavity. The mucous membrane lining of the tympanum was much thicker than natural, and in the upper osseous wall were observed a few small foramina for blood-vessels, and a carious orifice, of a size sufficient to allow the passage of a small pin. It is most probable that the disease of the tympanic cavity was the effect of that in the external meatus and in the lateral sinus.

